# Totally Laparoscopic Anatomical Hepatectomy Exposing the Major Hepatic Veins from the Root Side: a Case of the Right Anterior Sectorectomy (with Video)

**DOI:** 10.1007/s11605-014-2538-9

**Published:** 2014-05-20

**Authors:** Goro Honda, Masanao Kurata, Yukihiro Okuda, Shin Kobayashi, Katsunori Sakamoto, Keiichi Takahashi

**Affiliations:** Department of Surgery, Tokyo Metropolitan Cancer and Infectious Diseases Center Komagome Hospital, 3-18-22 Honkomagome, Bunkyo-ku, 113-8677 Tokyo Japan

**Keywords:** Laparoscopic hepatectomy, Anatomical hepatectomy, CUSA

## Abstract

Laparoscopic hepatectomy has rapidly evolved recently; 1–5 however, laparoscopic anatomical hepatectomy has yet to become widely used, although anatomical hepatectomy is ideal, especially for curative treatment of hepatocellular carcinoma, and is widely accepted via open approach. 6–10 This is because good-experienced skills, for example, exposing Glissonean pedicles and hepatic veins on the cutting plane, are required in order to perform anatomical hepatectomy via a pure laparoscopic approach. We obtained good results for various totally laparoscopic anatomical hepatectomies using the standardized techniques. We exposed the major hepatic veins from the root side by utilizing the unique view from the caudal side in the laparoscopic approach, and moved CUSA from the root side toward the peripheral side to avoid splitting the bifurcation of the hepatic vein. 11–13 We performed totally laparoscopic anatomical hepatectomy for 47 patients from August, 2008, to December, 2012 (Table 1). In most types of anatomical hepatectomy, the mean blood loss was <500 ml. Conversion to open surgery was required in two patients. Postoperative complications were prolonged ascites in two, peroneal palsy in two, and biloma in one. Mortality was zero. The embedded video demonstrates totally laparoscopic right anterior sectorectomy. In conclusion, our standardized techniques make laparoscopic anatomical hepatectomy more feasible.

**Table 1:** The result of 47 patients who underwent totally laparoscopic anatomical hepatectomy

	Number of cases	Time (mean)	Blood loss (mean)	Additional procedures	Conversion to open surgery	Complications
Rt. Hemi.	4	6 hr. 10 min.	270 g	Colectomy × 1, Stoma closure × 1	0	
Lt. Hemi.	4	5 hr. 06 min.	246 g	S5 partial × 1	0	
Rt. Ant. Sector	5	7 hr. 03 min.	596 g		0	Ascites × 2
Rt. Post. Sector	7	7 hr. 32 min.	382 g	S8 partial × 2, Rt.adrenectomy × 1	0	Peroneal palsy × 1
Lt. lateral Sector	7	3 hr. 29 min.	211 g		0	
Lt. Medial Sector	4	5 hr. 10 min.	310 g	S8 partial × 1	0	
Dorsal Rt. Ant. Segment	1	6 hr. 35 min.	395 g		0	Peroneal palsy × 1
S2 (segmentectomy)	1	7 hr. 15 min.	310 g	S4 partial	0	
S3 (segmentectomy)	1	3 hr. 22 min.	5 g		0	
S5 (segmentectomy)	3	6 hr. 28 min.	262 g		0	
S6 (segmentectomy)	4	5 hr. 00 min.	140 g		0	
S5 + 6 (segmentectomy)	2	8 hr. 14 min.	765 g		0	
S8 (segmentectomy)	2	8 hr. 00 min.	795 g		excessive time × 2	
Rt. Caudate lobe	2	8 hr. 51 min.	240 g	S2 partial & Coloctomy × 1	0	Biloma × 1

**Electronic supplementary material:**

The online version of this article (doi:10.1007/s11605-014-2538-9) contains supplementary material, which is available to authorized users.

This is a multimedia article. Please refer to the video included as a Supplementary Material.

## Electronic Supplementary Material

Below is the link to the electronic supplementary material.

ESM 1 (WMV 314,891 kb)
